# Elevating the field for applying neuroimaging to individual patients in psychiatry

**DOI:** 10.1038/s41398-024-02781-7

**Published:** 2024-02-10

**Authors:** David R. Roalf, Martijn Figee, Desmond J. Oathes

**Affiliations:** 1 Department of Psychiatry, Perelman School of Medicine, University of Pennsylvania, Philadelphia, PA, USA; 2 Neurodevelopment & Psychosis Section, University of Pennsylvania, Philadelphia, PA, USA; 3 Nash Family Center for Advanced Circuit Therapeutics, Icahn School of Medicine at Mount Sinai, New York, NY, USA; 4 Center for Brain Imaging and Stimulation, University of Pennsylvania, Philadelphia, PA, USA; 5 Center for Neuromodulation in Depression and Stress, University of Pennsylvania, Philadelphia, PA, USA; 6 Penn Brain Science Translation, Innovation, and Modulation Center, University of Pennsylvania, Philadelphia, PA, USA

**Keywords:** Neuroscience, Prognostic markers, Predictive markers, Psychiatric disorders

## Abstract

Although neuroimaging has been widely applied in psychiatry, much of the exuberance in decades past has been tempered by failed replications and a lack of definitive evidence to support the utility of imaging to inform clinical decisions. There are multiple promising ways forward to demonstrate the relevance of neuroimaging for psychiatry at the individual patient level. Ultra-high field magnetic resonance imaging is developing as a sensitive measure of neurometabolic processes of particular relevance that holds promise as a new way to characterize patient abnormalities as well as variability in response to treatment. Neuroimaging may also be particularly suited to the science of brain stimulation interventions in psychiatry given that imaging can both inform brain targeting as well as measure changes in brain circuit communication as a function of how effectively interventions improve symptoms. We argue that a greater focus on individual patient imaging data will pave the way to stronger relevance to clinical care in psychiatry. We also stress the importance of using imaging in symptom-relevant experimental manipulations and how relevance will be best demonstrated by pairing imaging with differential treatment prediction and outcome measurement. The priorities for using brain imaging to inform psychiatry may be shifting, which compels the field to solidify clinical relevance for individual patients over exploratory associations and biomarkers that ultimately fail to replicate.

## Introduction

Recent articles in high profile journals using large human samples have cast doubts on the role of neuroimaging in psychiatry. A recent study of >800 patients and >900 healthy controls found minimal evidence for a depression abnormality using structural MRI, diffusion-tensor imaging, and task-based or resting-state functional MRI [1]. Casting a wider net investigating behavioral measures relevant to psychiatry, a study of >1k students failed to find associations between structural MRI measures and the well-established ‘Big 5’ traits measured with the NEO-PI-R [2] thought to represent robust personality dimensions [3]. In another study of >50k individuals, brain-wide associations with cognitive or psychopathology-related measures did not manifest until thousands of individuals were included in analyses [4]. In addition to increasing sample sizes for analyses fostered by sharing data, there are other worthwhile approaches to make imaging more relevant to psychiatry and to psychiatric patients as individuals. Throughout this review, we discuss how imaging might inform decisions at the individual patient level and especially according to priorities set by patients themselves. A consideration not often discussed among imaging papers in psychiatry is the promise of ultra-high field (UHF) MRI which we discuss below. There is also a specific application of neuroimaging relevant to psychiatry that deserves special attention: combining brain stimulation with neuroimaging in psychiatry research. UHF imaging can offer more precision at the individual level whereas brain stimulation applications are particularly well-suited to applying imaging to an intervention also at the individual patient level. These separate or combined methods are well poised to “elevate” the science and clinical application of neuroimaging in psychiatry. Our discussion of integration can only be cursory, given the state of the field, but the immediate path forward for the methods individually and together are highly promising. Both methods are available now and can be better utilized to elevate the role of imaging for relevance to the practice of psychiatry. We conclude with suggestions for supporting paths forward towards impact at the individual patient level in psychiatry for the future.

### From high-field to ultra-high field MRI

Neuroimaging has evolved over the past several decades from a set of obscure methods applicable to small samples in highly specialized research centers to approaches that are ubiquitously available, sophisticated, and safe that provide multimodal parameters of brain structure and function. Specifically, 3 T MRI has become a stalwart tool in psychiatric research largely due to improved signal-to-noise ratio (SNR) from increasing magnetic field strength from 1.5 T. As a result, thousands of 3 T MRI studies are now published in many, if not all psychiatric conditions. MRI studies to date have expanded and broadened our understanding of how the brain generates and regulates behavior, but in general these advances have yet to directly benefit individual patients. Below, we briefly discuss currently available approaches for improving the specificity of 3 T MRI in psychiatry and then pivot to the future of imaging in psychiatry that will utilize UHF MRI approaches that will bring the field of imaging in psychiatry closer to benefiting individual patients.

One challenge for psychiatric neuroimaging is to find ways to integrate multimodal information into a coherent model that describes normative brain-behavior connections while realizing the aims of personalized medicine. The push in recent years to amass large amounts of MRI data for collaborative analytical efforts is a step in the right direction for elucidating biological evidence relevant in psychiatric conditions at scale [5]. But this must be complemented with mechanistically driven studies that allow a unique perspective on individual differences and offer insight into potential treatment. Examples of the latter include, but are not limited to, pharmacological functional MRI (phMRI) [6, 7] and stimulation protocols, before or during MRI. phMRI can be used to target receptor systems using specific drug compounds. Such studies have reported consistent and reproducible changes in relevant functional networks in disease and can detect dose-dependent functional network changes over the short and long term [8]. Recent phMRI studies have targeted neurotransmitter systems, specifically brain glutamate [6, 7], and serve as an example for parsing our understanding of heterogeneity within clinical conditions and response to treatment. For example, a phMRI investigation examining neural circuit functioning in chronic stable patients with schizophrenia during working memory fMRI found significant changes in striatum and anterior cingulate activation patterns that were attributable to a positive allosteric modulator of mGluR2 [7]. Notably, the inclusion of phMRI measures allowed the investigators to target known brain locations affected in schizophrenia, critical for working memory and high in mGluR2 receptor distribution to identify drug effects at the brain level. This specificity allowed the investigators to better understand potential heterogeneity in drug response and suggest the potential of increased mGluR2 signaling to improve disabling symptoms of schizophrenia [7]. More globally, the development of phMRI and other molecular imaging biomarkers can reveal therapeutic mechanisms, which will allow drug development and treatment to be tailored toward specific neural circuits and patient populations. Likewise, the use of noninvasive stimulation protocols (e.g., repetitive transcranial magnetic stimulation) in conjunction with 3 T MRI, in particular with magnetic resonance spectroscopy, can also provide convergent mechanistic evidence. As an example, systematic within-subject studies in depression indicate substantial change in neurometabolites, including glutamate and GABA, after short and long-term treatment with repetitive transcranial magnetic stimulation (rTMS) [9]. Larger, longitudinal studies where neural stimulation is coupled with 3 T MRI measures of both neurometabolism and network activity will provide a refined understanding of the influence neural stimulation has on excitatory and inhibitory neurometabolites and may offer support for a potential mechanism of action of this and other treatments. Moreover, it will enable the field to better map trajectories of change with treatment. Approaches like these will enable the field to evaluate early signals of clinical efficacy and build a roadmap for predicting clinical effects, all of which requires greater understanding of both disease and treatment effects on specific neural circuits. These efforts can be employed now at 3 T MRI and will narrow our path toward the goal of personalized medicine with optimal outcomes for every patient. While improvements in 3 T MRI approaches will improve incrementally in the years to come, which improve our ability to relate neuroimaging to behavior [4], and link improvements to measures of psychopathology, it is likely that the utility of 3 T MRI in psychiatry has reached its asymptote. Yet, other imaging opportunities exist and demand our attention; in particular the prospect of increased MR field strength for generating new discoveries in psychiatry.

FDA approval of 7 T MRI scanners for clinical diagnostic use in 2017 provides hope that ultra-high field imaging will improve sensitivity and specificity in psychiatric biomarkers and be applied in tracking clinical course and treatment response. Importantly, 7 T MRI in humans is well-tolerated [10] and produces higher functional sensitivity in clinical studies, including pre-surgical planning [11] as well as improved anatomical specificity in Alzheimer’s disease [12], Parkinson’s disease [13], multiple sclerosis [14, 15], epilepsy [16] and brain lesions [17, 18]. UHF MRI provides a significant increase in signal-to-noise ratio (SNR) and contrast-to-noise ratio (CNR), which confers near universal benefits compared with existing structural and susceptibility-weighted imaging. Moreover, reproducibility, reliability, and efficiency of measures like functional MRI are significantly improved at 7 T [19, 20] For example, topographic maps of function derived from 7 T MRI were more reliable, required far less data and had four times the predictive power as compared to using the same approach at 3 T [21]. In addition, the test-retest reliability of brain network mapping is significantly improved using 7 T versus 3 T [20], which bodes well for the use of 7 T fMRI. Specifically, within and between network connectivity measures were higher using fMRI data from 7 T and the reliability across subjects, who were scanned four times over six months, was significantly higher than at 3 T [20]. The noted improvement of UHF and its implementation in other disorders has paved the way for implementation in psychiatry. In the context of brain stimulation treatment in psychiatry, particularly, concerns with reliability and validity/artifacts with image-guided brain stimulation [22–26] might be expected to be mitigated using UHF imaging.

Ultra-high field studies are already extending our understanding of brain structure and function in psychiatry, which solidifies working knowledge across many fronts [27]. For example, a super resolution (400 mm^3^) structural UHF MRI study of the ventral tegmental area found that patients with anxiety and depression have reduced structural integrity of this dopaminergic structure compared to healthy individuals [28]. In addition, UHF studies have shown structural changes within subfields of the hippocampus [29] and insula [30] linked to improvement in depressive symptoms following treatment. UHF provides anatomical detail that cannot be realized at lower fields, which may make brain-behavior associations more feasible even with smaller sample sizes. For example, detailed anatomy of distributed functional networks, such as the default mode network, are better resolved at 7 T, which allows for more precise mapping within cortical gray matter [31]. The use of UHF fMRI has also revealed mood-related neurocircuit disturbances in patients with major depression not detected with 3 T fMRI [32]. These are just a few examples of recent studies where UHF MRI provides more nuanced views of global-level network disturbances in psychiatric conditions [27, 33, 34], but many of these applications remain diffuse and lack modifiable or druggable targets and, more work is needed to improve clinical utility.

**Fig. 1:**
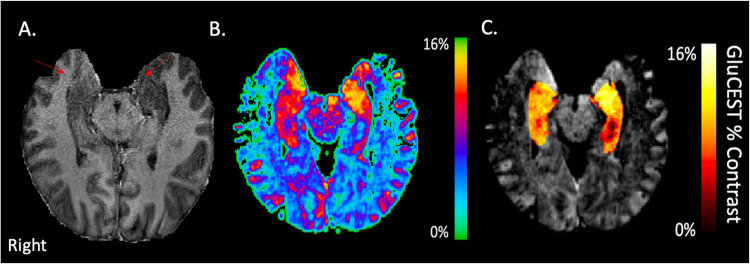
UHF Glutamate Chemical Exchange Saturation Transfer Imaging (GluCEST). UHF allows for high-resolution structural imaging (**A**) of the regions such as the hippocampus (red arrows). GluCEST imaging capture brain glutamate levels across the cortex (**B**) and regional analysis of glutamate levels in specific brain regions, such as the hippocampus, can be directly measured (**C**). Data show in radiological convention. GluCEST levels are shown in percent contrast levels [151]. Higher values are indicative of more glutamate concentration.

UHF provides improvements in ^1^HMRS and allows the application of novel approaches—such as chemical exchange saturation transfer (CEST) imaging [35]—both of which provide exquisite measurement of brain neurometabolites. Accurately assessing neurometabolic function is particularly relevant in psychiatry since there are several notable modifiable targets. It is well known that brain tissue contains substantial levels of glutamate and GABA—measurable without UHF [34]. While differentiation of cytoplasmic from synaptic neurometabolites remains challenging, the ^1^HMRS literature, particularly at UHF, provides evidence that these approaches detect psychiatrically relevant changes in neurometabolites. UHF ^1^HMRS studies in psychosis have provided convergent evidence of lower brain glutamate [36] and emerging evidence of disruptions in glutathione. Higher levels of glutathione have been associated with quicker response to antipsychotics, while higher glutamate is associated with greater impairment [37]. Convergent evidence using UHF GluCEST imaging and ^1^HMRS [38] further emphasizes glutamate as a target for intervention (see Fig. 1). Likewise, UHF ^1^HMRS studies in depression report alterations of glutamate and glutathione (relative ratios to creatine) [39] associated with some, but not all pharmacological treatments [40]. Collectively, these studies support the use of UHF ^1^HMRS and CEST as approaches that provide clinically relevant evidence that neurometabolic targets are targetable with interventions (including but extending beyond brain stimulation). ^1^HMRS and CEST at UHF are a step towards providing specificity in understanding multiple new biomarkers in neuropsychiatric disease.

Using group-level studies and emerging analytical approaches [41], it is possible to implement targeted investigations of individual patients using UHF. First, dimensional approaches at UHF provide measures tailored to an individual, particularly when existing circuit level knowledge is harnessed. For example, UHF GluCEST contrast in the brain’s reward network is dimensionally related to diminished reward responsivity in a transdiagnostic sample [42]. Notably, this approach links individual differences in reward responsivity to differences in neurochemistry within brain regions involved in reward encoding and valence evaluation. This study also used meta-analytically derived reward regions to increase sensitivity of GluCEST. This type of approach is an example of balancing more noisy individual data with group-level imaging data to aid in interpretation and possible utility at the individual level. While dimensional studies are a step in the right direction, there also needs to be a pivot towards more dense sampling of circuits, systems and individuals. Examples of approaches that densely sample individuals at 3 T MRI [43] could be implemented at UHF with greater precision. For example, subdivisions of the amygdala have been identified using individual functional connectivity networks at 3 T [43]. A recent study used a similar approach in two individuals with depression at UHF [27]. Here, functional network parameters were measured before and after treatment in hopes of determining whether a particular therapy (electroconvulsive therapy or medication) had measurable effects on individual networks. This preliminary application demonstrates potential for precision psychiatry, particularly to identify which treatments may be optimal for a given individual. Not only can this type of approach be implemented at UHF, it can be extended to neurometabolite levels (e.g., glutamate) which will likely allow for better parcellation and precise therapeutic targets. Mapping glutamate levels among functional networks could help elucidate patterns of network-specific hyper- and hypo-excitability in psychiatric disease states. This lays the groundwork for individualized assays of function (or dysfunction) in individual patients. By implementing UHF protocols that use repeated-dense sampling and precision parcellation, personalized outcomes are feasible. The broader field of MRI is moving towards individualized assessment of network disturbances. There is evidence that individual-specific functional network topography in neuropsychiatric conditions is measurable [41] and that understanding functional topography may be critically important for understanding neurodevelopmental substrates (e.g., glutamate) of these disorders. Individual profiles of functional connectivity or neurometabolite distribution represent signatures for each patient also sensitive to state changes. The challenge is to bring these functional or neurometabolic assessments into alignment with clinical assessment to provide individualized treatment options and prediction.

There is at least one recent implementation of convergent UHF and TMS that could be a roadmap towards precise psychiatric evaluation [44]. Specifically, UHF GluCEST has recently been used to study effects of TMS in healthy individuals [44]. This study found that GluCEST signal decreased following continuous theta burst rTMS. The decrease was not limited to the target site of stimulation, but rather diffuse within the imaging slice. Notably, both the stimulation site and downstream brain regions could be simultaneously measured. If this had been attempted with ^1^HMRS, the spatial distribution would have been entirely missed given limited spatial coverage. 3 T ^1^HMRS studies also show promising changes in brain neurometabolies to TMS [9, 45] that can be extended at UHF. Given these initial results, future studies combining TMS and GluCEST will contribute to understanding of metabolic changes associated with TMS which should aid in developing precision approaches in individual patients. UHF offers opportunities to amass comprehensive data to disambiguate endogenous disease-related alterations from treatment-induced effects and to characterize how different treatment approaches affect brain neurometabolites, both during initial phases and after long-term treatment.

### Summary

The direct measurement of in vivo brain neurometabolites in humans is challenging but by taking advantage of advances in UHF MRI, progress can be made at the cutting edge of psychiatry.

As 7 T MRI becomes more common and is applied to more neuropsychiatric conditions, the field should consider establishing UHF consortia with standardized acquisition and analysis guidelines, similar to the European Ultrahigh-Field Imaging Network for Neurodegenerative Diseases (EUFIND) [46]. The growing availability of publicly available 7 T MRI data [47] offers analytical opportunities to hone data-driven approaches that will refine our understanding of the intricacies of brain function and provide a basis for interpretation of functional abnormalities in clinical samples. Access to 7 T MRI remains somewhat limited in North America, but the number of 7 T MRI scanners has grown rapidly over the past decade and there are now 36 operational 7 T MRI scanners. Given the approval of 7 T MRI by the FDA, the number of 7 T scanners will only continue to grow and improve access for both research and clinical use. Clearly, improving access will be a vital step toward reducing methodological heterogeneity across protocols and improving neuroimaging measures for applications in psychiatry.

### Brain stimulation as a special case

In addition to generating more precise brain signals and exploring a wider variety of brain image-derived measures, psychiatric relevance may also be proven by better matching research and clinical questions in terms of specific brain circuits or networks. Brain stimulation as routinely applied transcranially through direct current, alternating current, or electromagnetic methods pre-selects individual and specific stimulation targets at which to deliver stimulation intended to induce neuromodulation. Deep brain stimulation (DBS) likewise necessarily plans a locus where the stimulating electrode will be implanted in the brain for remediating symptoms of neurological and psychiatric patients also through neuromodulation (as opposed to ablation). Seizure therapies are highly efficacious and methods are advancing but given a lack of focality for the seizure event, are not further discussed here. Studies of brain injury (including ablation) can inform knowledge of mechanisms when injuries impact psychiatric symptom presentations but are also outside the scope of this review. Instead, we focus on the multi-faceted relevance of neuroimaging as a brain-based measure in the context of brain stimulation as a brain-based intervention. It may be argued that all of neuropsychiatric treatment is ‘brain-based’ given that we expect effective treatments to change the brains of patients we treat. However, the priority given to a specific brain target or pathway in the context of brain stimulation treatment is unique compared to broader targeted pharmaco- and behavioral therapies. These targets are by necessity applied at the individual patient level though the degree of personalization varies tremendously across clinical and research applications. Given the unique **convergence** between **measurement** and therapeutic **intervention**, we believe that brain stimulation offers an ideal proving ground for demonstrating the relevance of neuroimaging to psychiatry. Below we highlight promising applications of neuroimaging to brain stimulation in psychiatry then provide suggestions to further build this promising field. Given a clinical relevance priority in this review, we also discuss applications and hurdles for applying methods at the individual patient level.

### Group-level imaging applications for discovery and symptom mapping

DBS and TMS studies utilizing normative datasets (sometimes called ‘connectomic’ but this term also has broader applications), aggregated from group averages, explore network-level [9] understanding in patient responses to treatment based on which networks were stimulated in template space [48–53]. This approach can increase understanding of clinical response variability in aggregate. For example, normative connectivity maps suggest that invasive and non-invasive effective treatments target similar brain networks [54]. Following evidence that lesions in distal brain regions can cause the same symptoms when they overlap in the same normative brain network, lesion mapping has been applied across many neurological and psychiatric symptoms, deepening our understanding of how symptoms may be caused and potentially treated [55]. An early study on brain-symptom biotypes in depression found differential responsiveness of patient subtypes to rTMS treatment at a single target [56] which underscores (sub)group-level data with clinical potential for refinement and development. More recently exploring differential symptom responses to rTMS across standard-space functional connectivity defined networks from a large healthy participant atlas, a study of depressed patients suggested specific symptom clusters that differentially respond to TMS treatment depending on which network is stimulated [50].

This work is highly relevant to decisions made at the individual patient level given it supports the theoretical view that brain-based interventions can be targeted to specific symptom clusters. However, these approaches do not prioritize explicitly which node within a network should be targeted for a particular patient suffering from a particular symptom. To form this bridge for individual patients, prospective patient-specific targeting and randomized target assignments are essential. Aggregating data on which symptoms respond to stimulation will likely play an important role in tailoring brain-based interventions to individual patients. Using normative/group data to constrain brain stimulation targeting would likely mitigate confounds with individual imaging data that can generate false-positive maps modeled on non-neuronal noise. The degree to which an individual brain signal converges with ‘known’ patterns in group data relevant to that same function is suggestive of a robust and valid brain target at the individual level. Though normative stimulation targets may not always improve clinical outcomes [57], the relative ease of targeting using brain atlases is driving their further exploration [58] especially in the clinic.

Whether normative data are sufficient or whether individualized markers warrant resource demands (scan costs, experts to process data) likely depends on the complexity of the neurobiology targeted and the effect size difference between normative and individual targeting treatment outcomes. In Parkinson’s disease, for example, differences between normative and patient-specific brain connectivity from easier to define gray matter DBS targets (subthalamic nucleus, globus pallidus) may be smaller [59] than in many psychiatric disorders that may need more individualization given that targets are located at critical junctions of white matter networks (internal capsule, subcallosal cingulate-SCC) that are complex and variable [60, 61].

Among proponents of normative connectomic atlases for symptom/brain pathway integration, there is recognition that fine-tuning based on individual imaging data is likely to be of value in some cases [62]. Normative databases do not explicitly account for variability at the individual patient level. In one study using an atlas-based target for treating depression with rTMS, which was individualized based on patient structural (not functional) MRI, did not yield better efficacy compared with a traditional scalp-based target [57]. There was also a failed trial treating bipolar depression with the same group-based target [63] as well as a failure of a structural MRI defined target (Heschl’s gyrus) in treating auditory hallucinations in schizophrenia [64]. There are likely *some* scalp-based targets that do not do as well as *some* structural MRI coordinates [65] given that one of these may overlap better a brain network instrumental to clinical effects. There are pitfalls with finding the *right* atlas-based map. In addition to the well-characterized caveat about low reliability in imaging derived maps over time in the same individual [66], another concern is that group average data can be skewed by a minority of participants showing particularly strong activation at a particular voxel. This average map then does not reflect where the largest *proportion* of participants had fairly strong circumscribed activation [67]. This shortcoming compounds the group-to-individual mapping problems described as symptom heterogeneity within psychiatric disorders [68], within patients themselves fluctuating symptom-wise over time [69], and in group-average fMRI data biased by a subset of extreme individual values that may not correspond to many or even most patients in a group [22, 67, 70, 71]. For individual patient imaging data to be clinically useful, all of these concerns must be effectively addressed. In the case that we have hypotheses that neuroimaging data from a particular patient could be useful to guide/refine a targeted brain stimulation intervention, there may be a number of viable options.

### Strategies for targeting individual patient stimulation

In our work, we have seen differential circuit engagement to brain stimulation using the same fMRI-guided targeting for depressed compared with healthy participants [72] and another study found a lack of amygdala engagement to TMS in MDD patients present in healthy subjects [73], stressing that normative network maps from healthy individuals may not agree with patient brain maps. Within diagnostic groups, symptoms, brain clusters, and demographic variables are known to exist and influence differential responsiveness to brain stimulation treatment [56, 74–80] suggesting further attention to variability at the individual level. In an influential early study comparing behavioral effects based on TMS targeting approaches, the strongest effects were found using individual fMRI-guided stimulation followed by MRI-guided, followed by group atlas-based coordinates and with substantially weaker effects using a scalp-based target from EEG [81]. In post-hoc analyses, proximity to an individual FC peak is associated with better treatment response to rTMS in depression [82–84] even when statistically controlling for proximity to the group average target [84]. Prospective targeting with individualized FC for rTMS treatment of depression has shown to be the most effective treatment reported in the literature to date for treatment-resistant depression [85–87] though the rTMS protocol itself was also substantially different from prior trials. Similarly, fMRI from a gambling task was used successfully to treat (71% symptom reduction) generalized anxiety disorder [88] and that coordinate was used to successfully treat patients in a subsequent study which showed differentiation from sham and also brain changes correlated with symptom improvement in GAD [89]. The group coordinate target was less effective in improving symptoms (52% on the same [Hamilton] anxiety scale). For rTMS in depression, there may be a relationship between how close the individual peak EEG alpha frequency is to the standard 10 Hz frequency used to treat patients [90] though it is unclear if changing the standard frequency to match patient peaks increases efficacy.

**Fig. 2:**
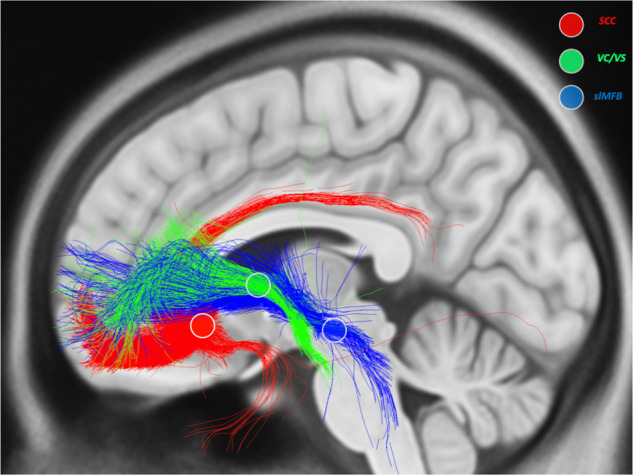
Three Common Pathways for Psychiatric Deep Brain Stimulation. Stimulation target (circles) and activated connections with DBS in subcallosal cingulate (SCC, red), ventral capsule/ventral striatum (VC/VS, green) and superolateral medial forebrain bundle (MFB, blue). Connectivity profiles from each target generated using normative group connectome diffusion tractography data from Lead DBS software. Figure made by Ki Sueng Choi.

Neurosurgical approaches implant electrodes in subcortical brain regions for neuropsychiatric applications and though patient groups are small, differential outcomes associated with variations in targeting can lead to understanding about which brain targets improve which symptoms. Historically, DBS targets have been identified pre-surgically on structural MRI scans in standard stereotactic anatomical space based on atlases [91, 92], which fails to consider individual variability of gray matter morphology or white matter fibers. More recently, advances in diffusion tensor magnetic resonance imaging (dMRI) tractography and tissue activation models highlight the emerging role of stimulating individual white matter networks in DBS for psychiatry. Riva Posse and colleagues performed a retrospective analysis on patients treated with SCC DBS (Fig. 2) using individual activation models and probabilistic tractography [93]. The response rate to SCC DBS was 58% and all responders shared a common map of stimulated white matter fibers. In their subsequent SCC DBS cohorts, surgical targets and contacts for chronic stimulation for each patient were based on this tractography map, resulting in substantially improved response rates of 82% and 88% [94]. Another DBS target for depression, the medial forebrain bundle (MFB), has always been based on individual tractography as the MFB is not visible in standard atlases (Fig. 2). Response rates in trials with tractography-based MFB targeting average 70% [61], were again superior to observed outcomes with non-diffusion MRI-based targets. The anterior limb of the internal capsule (ALIC) is a DBS target for both OCD and depression. A retrospective study in 50 OCD patients treated with ALIC DBS used normative connectomic data to model which ALIC fiber stimulation was most correlated with improved obsessive-compulsive symptoms [49]. The OCD responsive tract consisted of connections between thalamus, brainstem, and ventrolateral and medial prefrontal cortex, which was then replicated in a separate patient cohort by an independent group [95] and has since been replicated by multiple other centers. The hope is therefore that prospective studies using tractography-based surgical planning and parameter optimization in ALIC DBS will demonstrate substantially improved outcomes and predictability over traditional targeting.

Beyond reproducible and anatomically precise DBS targeting, neuroimaging studies will also be essential in understanding *how* stimulation of specific pathways influences heterogeneous symptoms. In OCD, it was shown that OCD symptoms were effectively remediated with electrode placements either in the anteromedial subthalamic nucleus (amSTN) or ventral capsule/ventral striatum (VC/VS; Fig. 2) [96]. Both targets improved depression symptoms though VC/VS moved them more. By contrast, only the amSTN significantly improved cognitive inflexibility. These pathway-symptom response profiles could be of relevance for invasive and non-invasive neuromodulation across diagnoses. In depression, SCC DBS primarily improves negative affect, in accordance with the primary mood regulatory function of the SCC and its connections to structures related to interoception such as the insula and cingulum [21]. On the other hand, patients responding to DBS of the VC/VS or MFB, targets more connected to regions associated with reward and positive affect (Fig. 2), acutely improves anhedonia and motivation for pleasurable activities [97].

To maximize the utility of individualized functional imaging-based brain targets, it is important to demonstrate that a target is viable as a therapeutic focus even in the context of variability or noise inherent to individual imaging data [22, 25, 98]. Noise, spurious connections and transient physiological fluctuations may contaminate reliability of highly individualized brain maps. This has been explored in resting fMRI connectivity-based targeting for the subgenual anterior cingulate cortex as a depression target in TMS [22]. Improved imaging protocols [23], higher field strength MRI and improved processing steps [22, 99, 100] can further improve SNR as well as reliability of these targets. In addition, steps to constrain individual data with group-wise imaging databases may improve reliability and especially relevance to psychiatric pathology. Weighting individual FC by a group average [22] and selecting consistent clusters as opposed to peak voxels [99] have been used to address fluctuations in connectivity intra-individually. Prioritizing stimulation targets that fall within spatial boundaries generated from group atlases of ‘depression’ or ‘PTSD,’ etc. available as search terms in Neurosynth [101] or Neuroquery [102] might be additionally effective in balancing individual and group-level evidence. Likely the most informative group-level priors for constraining individual maps should be based on brain stimulation targets that improve specific symptoms [50] according to the primary complaint of the patient. Expanding the evidence base for which pathways respond to brain stimulation and particularly influence specific symptoms is essential evidence that will allow clinicians to respond to the needs of patients, prove the utility of imaging data for this application and ultimately finally influence the actual practice of psychiatry using brain imaging data.

### Learning from concurrent brain imaging/ stimulation

Just because a white matter tract is observed on a DTI image, a resting fMRI correlation between brain areas is observed, or a brain area shows increased BOLD signal to a task does not in and of itself mean that stimulating one of these brain areas will engage the intended network. Even with impressive clinical effects from fMRI-guided brain stimulation [86, 88], the link between circuit engagement or modulation to brain stimulation that could explain variability in clinical responses remains a black box without also adding individual patient brain recordings [73, 89, 103, 104]. More broadly in pursuit of brain/behavior linkages, the relative importance of specific network nodes and their ability to control the network is not easy to prove without causal and acute manipulation of that node. Many factors including local and distributed connections from the stimulated brain area as well as the brain state of the stimulated area and network at the moment of stimulation affect the ability of a node to engage specific pathways or networks. For these same reasons, electric field models will always be limited in their ability to predict pathway engagement given fixed estimates of biological susceptibility to stimulation (though excellent for comparing coil designs assuming biological constants [105, 106]). Traditional imaging measures also do not easily inform directionality in circuit communication [107].

**Fig. 3:**
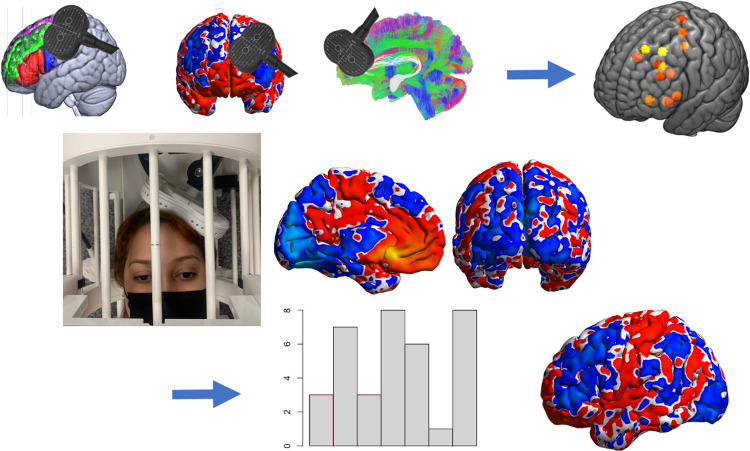
Brain Stimulation During Imaging for Target Engagement and Symptom Improvement. In the First Row, several potential imaging data points for use in brain stimulation targeting are proposed and used to generate patient-specific maps of potential network targets (orange square). The targets are stimulated iteratively during concurrent brain imaging (Second Row) to generate full-brain evoked brain responses for each stimulation site. These are used to validate the optimal network targeting approach in the First Row. An intervention is then applied and symptom changes are associated with brain circuit engagement, pre-intervention, to generate a new map of circuit engagement by symptom change (Third Row). This then becomes the new target for intervention.

Adding imaging before and after an intervention is a useful demonstration that brain activity does indeed change to the intervention and these changes can be linked to symptom changes in a way that sets up hypotheses for subsequent targeted brain stimulation interventions [108] (Fig. 3). For example, if the changes in downstream brain areas not specifically targeted with stimulation are most associated with a desired clinical effect, it would be reasonable to try a *subsequent intervention* focused on these neural pathways. Acute brain responses to stimulation can also be captured and tested for their ability to predict clinical effects at the patient level [108–112]. ‘Circuit engagement’ associations with clinical effects are especially relevant to brain stimulation interventions but may also inform circuit integrity predictors of other interventions, such as psychotherapy [109], provided that the engaged circuits mechanistically contribute to therapy effects. The directionality in causal circuit communication testing with these methods allows a distinct advantage compared with brain imaging alone. When brain stimulation is done during brain imaging data acquisition, technical challenges are rewarded with definitive knowledge of how brain circuits are engaged at the individual patient level [108, 110, 112–114].

In psychiatric populations, there are a handful of concurrent DBS/fMRI studies in OCD and depression. While OCD studies have not yet shown a relationship between circuit engagement and clinical outcome [53], the result of stimulating the sgACC in depression has produced clinically relevant findings. DBS-evoked fMRI signal in the dorsal anterior cingulate cortex (dACC) correlated with acute mood fluctuations in the scanner and also predicted long-term depression improvement [115]. With recent DBS devices it has become feasible and safe to combine 3 T fMRI with active DBS to directly examine network effects of DBS as a potential biomarker for parameter optimization. Using 3 T fMRI in patients with STN DBS for Parkinson’s disease, a recent study identified a reproducible pattern of brain response to optimal DBS parameters marked by preferential engagement of the motor circuit [116]. Objective neuronal markers of DBS efficacy would be especially helpful in psychiatric DBS where reliable acute clinical responses are not typically observed. UHF metabolic imaging and fMRI in response to DBS can be invaluable tools to indicate brain responses during DBS engagement. In DBS, these neuronal markers can also be captured uniquely with electrophysiological recordings from implanted leads.

In two proof-of-concept N-of-1 studies, such intracranial recordings demonstrated an individual pathway and signal for targeted deep brain stimulation to effectively treat depression [117, 118]. Extended recordings with intracranial leads informed by natural or experimental symptom provocation are a gold standard in determining brain stimulation targets. While we can learn a great deal from these patients, data are acquired at a very low rate given the resources required and invasiveness of the recordings. A middle ground that can be applied to nearly every patient is to pair non-invasive brain imaging with non-invasive brain stimulation.

Interleaved (aka ‘concurrent’) TMS/fMRI allows for both circuit mapping (single pulse evoked brain response) and neuromodulation investigations to be conducted in the scanner while brain responses are captured [108, 112, 113, 119–122]. TMS/fMRI can be used to test basic circuit and network interactions with causality inference usually only afforded by invasive methods [108, 110]. It also demonstrates circuit engagement critical in exploring activity propagation only imperfectly measured using resting fMRI correlation, DTI, etc. Both resting fMRI [112, 113, 122–124] and DTI [125] have been used to target cortical-subcortical pathways, evidenced by interleaved TMS/fMRI, which opens up treatment targets deep in the brain such as the subgenual anterior cingulate cortex [112, 123, 125] and the amygdala [122, 123]. Scalp based [119, 126, 127], structural anatomical [128] and template-based [73, 109, 129] targeting also have been used (non-exhaustive list; cf [110]) and generate deep and remote fMRI BOLD responses. Interleaved TMS/fMRI is an especially powerful method under development for demonstrating target engagement and modulation in the context of rTMS treatment [108, 110–112] (see Fig. 3). Though this is an emerging field, recent work suggests that reliability of the TMS-evoked fMRI BOLD response can be improved by averaging at least 50 single pulse stimulation ‘events’ per circuit probe [130] which we exceeded in our recent work [123] perhaps contributing to our success in replicating both subcortical evoked responses in larger subsequent samples [72, 112, 122]. The testing benchmark for novel TMS methods is the motor-evoked potential given the ease with which circuit engagement and modulation can be tested using surface electromyography of the downstream targeted muscle group. Methods focused on increasing reliability of MEP measurement may also increase TMS reliability with other outcome measures and have included adding sufficient recovery time between single TMS pulses (Inter-pulse interval), using neuronavigation to maintain the target, collecting more trials (20 + ), input-output stimulation level-MEP curves, and the choice of an optimally sensitive output (muscle) to detect induced changes [131, 132]. Applications to clinical and cognitive domains also suggest careful more individualized scalp targeting as well as manipulating or measuring state-dependent influences [133, 134]. Advances in equipment [135], scanning protocols, analysis methods, and targeting approaches [136, 137] can be combined to show relationships between brain-evoked responses, acute neuromodulation, and clinical outcomes [108, 110–112]. Though there is not currently a 7 T compatible TMS coil, pre/post rTMS MRI images at 7 T as well as UHF imaging predictors can add precision as well as mechanistic understanding of rTMS effects on the brain.

Though it is expensive to frequently sample MRI or PET, EEG can be acquired frequently without prohibitive costs. A recent study found that a TMS evoked EEG potential tracked with depression improvement to an rTMS intervention though it did not serve as a prognostic predictor of outcome from baseline [73]. In this study, resting fMRI (global FC) did predict treatment outcome and tracked with depression improvement (dorsolateral prefrontal FC). Another less expensive method, functional near-infrared spectroscopy, measures oxygenation changes associated with brain activity and has been used successfully to demonstrate dlPFC FC changes associated with depression improvement to rTMS [138]. Again, no prognostic predictors were identified.

#### Summary

Brain stimulation is a special case and a proving ground for neuroimaging utility for psychiatry for multiple reasons: the way rTMS is delivered in the clinic requires an inherent hypothesis for a specific brain region or pathway being critical for improving symptoms through neuromodulation. Concurrent imaging with brain stimulation is a rapidly growing field given its potential to yield mechanistic insights into how stimulation propagates through specific circuits for a specific patient and how this communication changes with treatment.

### Next steps in linking brain stimulation and imaging in psychiatry

There are unanswered questions on how imaging can inform brain stimulation but a variety of studies have taken first steps in how to address these questions [108, 110]. The field of concurrent imaging with brain stimulation is especially valuable for linking circuit engagement with outcomes to brain stimulation therapies. In addition, pre/post measures aggregated by circuits targeted, patient factors and symptom change will continue to be valuable and do not require specialized equipment (such as that for concurrent TMS/fMRI). Tracking what pathway was stimulated based on normative [50] (or even better, patient) databases can help to guide and further optimize treatment target selection. However, brain network representations identifiable at the individual level can easily get lost in group averages that by necessity require spatial co-localization in a standard space [70]. At the same time, idiosyncratic noise must be avoided which requires balance between normative and individual data.

The fidelity of intracranial brain recordings is ideal for measuring brain dynamics, symptom fluctuation related brain activity patterns, and brain responses to stimulation at acute and longer timelines. The high demand on resources and medical risks with invasive procedures to patients will continue to keep sample sizes low in these studies. As such, evidence from definitive invasive small patient studies should guide non-invasive imaging and stimulation interventions so that findings can generalize to nearly every patient participating in lower risk and lower cost non-invasive treatments. For example, an invasive recording of a patient during symptom provocation may yield a tractable signal associated with specific symptom elevations. In the same patient, iterative testing of various stimulation parameters and locations may yield a viable target for subsequent implant of a deep brain stimulator. In the same patient, resting fMRI may have also been collected and/or cortico-cortico evoked potentials during invasive recordings. This circuit level information may support a cortical pathway through which it would be reasonable to attempt a non-invasive trial in treating other patients with the same symptom elevations. UHF fMRI signal in the original patient or in the subsequent non-invasive imaging and treatment cohort may yield better maps of connectivity that correspond closer to cortico-cortical evoked potential data and are superior in guiding non-invasive stimulation targets.

### Next steps in neuroimaging for psychiatry

After decades of research, the field of psychiatry continues to struggle to demonstrate the relevance of neuroimaging for clinical practice. There are many hurdles to effectively using data as collected in typical psychiatric research imaging labs to inform clinical practice. These hurdles include: incentives for protecting and publishing small sample studies necessary for career advancement of scientists, image processing almost always unique to each laboratory, symptom fluctuations over time not captured by single imaging timepoints, heterogeneity within patient samples, lack of mechanistic understanding of treatments, lack of symptom-relevant experimental tasks/contexts, and other factors. Most imaging studies published in the recent past cannot address these shortcomings. At present, clinicians can be maximally effective in practice while ignoring entirely the field of neuroimaging. A focus on individual patient data is consistent with clinical practice but is also at least partially at odds with cognitive neuroscience approaches to understand how the brain works writ large. For example, sophisticated derived brain features can be reliable and show associations with latent behavioral traits but at the same time generate difficult to interpret models at the level of neuroscientific understanding [139] and are especially difficult to apply to individual patients.

Knowing that patients according to hippocampal volume, white matter intensity, amygdala activation, default mode and/or executive control network connectivity will or will not respond better to an antidepressant medication is noteworthy and supports the possibility of an eventual differential clinical response [140, 141]. However, this information is not immediately useful at the individual patient level since, no matter the individual patient MRI, comparisons of multiple treatment choices according to biotype are lacking leading the clinician to simply choose first line treatments for that patient group. This unfortunate course is driven by the lack of evidence that, for example, a patient with hippocampal volume greater than a given threshold will respond better to treatment A vs. other options. We need large studies observing patients randomized to treatments tracking outcomes based on imaging data so that imaging can prove its utility above the standard of care.

Personalizing treatment based on imaging is a worthy goal. However, it is worth considering that a behavior association with brain circuits is not sufficient to define a subjective mental state central to patient clinical complaints without explicitly linking these measurements [142]. If looking at fearful facial expressions or emotionally reappraising / reinterpreting pictures of car accidents indeed elicits the same feelings of distress or failed attempts at regulating distress that patients report as central to their reason for seeking treatment, those would be useful tasks to implement while measuring brain activity. On the other hand, if we are falling short of eliciting clinically relevant experiences with these common paradigms, more work is needed to increase the relevance of tasks acquired with imaging data to patient experiences. Symptom provocation has leverage now in rTMS treatment given it is part of the FDA approved intervention for OCD [143]. Yet, we do not actually know what happens at the circuit level, how important this particular provocation is for clinical outcome, or whether the responses to the provocation change with treatment. Symptom-related brain circuit investigation at the individual level is likely to further our understanding in aggregate at the group level, as well. Nonetheless, this evidence alone does not describe what circuits are actually engaged at the individual patient level which is likely critical to optimize individual treatment planning. An identical concern has been described even in basic motor-evoked potential neuromodulation studies that are the precursors of rTMS clinical interventions. At the individual level, there are numerous cases where neuromodulation is not successful [144] which, not surprisingly, occurs clinically [145, 146] and is poorly understood at the level of the individual patient. Evidence is building that group average neuroimaging data are not as clinically informative as individual brain imaging measures [84, 89]. Brain stimulation combined with imaging can be an especially effective proving ground for linking circuit engagement to circuit modulation and symptom improvement as a special case for a brain measure that is conceptually closest in relevance to the applied intervention.

Treatments broadly can be better understood and optimized through focused imaging measurement including UHF MRI that has a variety of new applications for understanding neurometabolic predictors and measures of brain changes to existing and emerging treatments including ketamine, computer-based psychotherapy and brain stimulation. Sensitive and reliable tools such as UHF and TMS can provide insight into the biological mechanisms underlying incipient psychopathology providing direction for appropriate use of pharmacological treatment. With increased availability of UHF, these new applications can make the leap from research to clinical use. Novel approaches, such as GluCEST, will allow for means to assess novel measures of function and dysfunction across the brain. Moreover, integrating UHF approaches with specific treatments (e.g., TMS, ketamine) that affect neurotransmitter systems is likely to provide insight into excitation-inhibition processes allowing for more targeted treatment in individual patients. For example, the application of a novel UHF ^1^HMRS approach, quantitative exchanged-labeled turnover MRS (qMRS), allows for the measurement of dynamic changes in brain glutamate and its derivatives, potentially providing an opportunity to measure glutamate-glutamine turnover [147]. As understanding of biology continues to improve, innovative MRS techniques, such as functional MRS, X-nuclear (^31^phosphorus or ^13^carbon) MRS, and dynamic testing (e.g., ketamine infusion during MRI scanning), may help identify new relevant biomarkers in vivo [34]. New information gained will enable novel applications of targeted treatment and provide mechanistic targets for improving the implementation of precision psychiatry.

Finally, we acknowledge that the integration of neuroimaging into the standard practice of clinical psychiatry faces hurdles and raises significant questions concerning incremental validity, utility, access, cost, and the robustness of findings from the extant neuroimaging literature. There are also potential professional issues, such as training, credentialing, and interdisciplinary boundaries to consider. Notwithstanding these considerations, we can envision psychiatry of the future where modern neuroimaging neuroscience provides adjunctive structural, functional or biochemical information to parse clinical heterogeneity. These additional readouts will include information on irregularities in neural structure, hyper or hypoconnectivity of personalized functional networks related to behavioral deficits, and/or elevations or reductions in brain neurometabolites, all of which can aid in the decision-making of future psychiatrists. Ultimately, the burden here relies upon collaborative efforts between psychiatry and neuroscience to elucidate these mechanistic links and provide readouts that are applicable and accessible to all clinicians.

Our recommendation for the field is 1) to combine active treatments in individual studies with larger samples, diverse patient groups, repeated samples and with symptom-relevant tasks. We also recommend that 2) the best imaging findings in psychiatry be translated into interventions and this includes emerging UHF applications. Neuromodulation experiments validate correlative imaging maps, can cause brain as well as behavior changes and can be 3) run with concurrent or pre/post imaging to understand mechanisms of effects across individuals. This evidence is a critical bridge for guiding novel brain-based therapies. We also support promising approaches to 4) improving symptom measures [148] as well as prioritizing social, academic, and vocational functioning [149, 150] in addition to mood or other dynamic processes in the search for neurobiological associations relevant to psychiatry. Finally, we suggest that the field 5) work to discover differential treatment prediction markers and evidence of neuroplasticity with imaging as a priority above basic biomarker studies or brain-wide association exploration that requires larger sample sizes and a less direct path to clinical application. In all of these suggestions, we prioritize generating imaging findings that are more precise and more useful at the level of the individual patient. This is the essential detail that will convince the clinical practice of psychiatry that brain imaging can, in fact, hold direct relevance for treating individual patients.
